# TripNet: A Method for Constructing Rooted Phylogenetic Networks from Rooted Triplets

**DOI:** 10.1371/journal.pone.0106531

**Published:** 2014-09-10

**Authors:** Hadi Poormohammadi, Changiz Eslahchi, Ruzbeh Tusserkani

**Affiliations:** 1 School of Biological Sciences, Institute for Research in Fundamental Sciences (IPM), Tehran, Iran; 2 Department of Computer Science, Shahid Beheshti University, G.C., Tehran, Iran; 3 School of Computer Science, Institute for Research in Fundamental Sciences (IPM), Tehran, Iran; SUNY Downstate MC, United States of America

## Abstract

The problem of constructing an optimal rooted phylogenetic network from an arbitrary set of rooted triplets is an NP-hard problem. In this paper, we present a heuristic algorithm called TripNet, which tries to construct a rooted phylogenetic network with the minimum number of reticulation nodes from an arbitrary set of rooted triplets. Despite of current methods that work for dense set of rooted triplets, a key innovation is the applicability of TripNet to non-dense set of rooted triplets. We prove some theorems to clarify the performance of the algorithm. To demonstrate the efficiency of TripNet, we compared TripNet with SIMPLISTIC. It is the only available software which has the ability to return some rooted phylogenetic network consistent with a given dense set of rooted triplets. But the results show that for complex networks with high levels, the SIMPLISTIC running time increased abruptly. However in all cases TripNet outputs an appropriate rooted phylogenetic network in an acceptable time. Also we tetsed TripNet on the Yeast data. The results show that Both TripNet and optimal networks have the same clustering and TripNet produced a level-3 network which contains only one more reticulation node than the optimal network.

## Introduction

Phylogenetic networks are a generalization of phylogenetic trees that permit the representation of non-tree-like underlying histories. A rooted phylogenetic network is a rooted directed acyclic graph in which no node has indegree greater than 2 and the outdegree of each node with indegree 2 is 1. Such nodes are called reticulation nodes. In rooted phylogenetic networks the nodes with indegree 1 and outdegree 0 are called leaves and are distinctly labeled by a set of given taxa. Mathematicians are interested in developing methods that infer a phylogenetic tree or network from basic building blocks. In the computation of a rooted tree or network, one group of the basic building blocks are rooted triplets, the rooted binary trees on three taxa [1].

In 1981, Aho et al., studied the problem of constructing a rooted tree from a set of rooted triplets [2]. They proposed an algorithm called BUILD algorithm which shows that, given a set of rooted triplets, it is possible to construct in polynomial time a rooted tree that all the input triplets are contained in it or decide that no such tree exists.

When there is no tree for a given set of triplets one may try to produce an optimal phylogenetic network. In this context, the goal is to compute an optimal rooted phylogenetic network that contains all the rooted triplets. One possible optimality criterion is to minimize the *level* of the network, which is defined as the maximum number of reticulation nodes contained in any biconnected component of the network. The other optimality criterion is to minimize the number of reticulation nodes [1]. In [3] and [4] the authors considered the problem of deciding whether, given a set of rooted triplets as input, is it possible to construct a level-1 rooted phylogenetic network that contains all the input triplets? They showed that, in general, this problem is NP-hard. However, in [4] the authors showed that when the set of rooted triplets is dense, which means that for each set of three taxa there is at least one rooted triplet in the input set, the problem can be solved in polynomial time. After their results, all research in this new area has up to this point focused on constructing rooted phylogenetic networks from dense rooted triplet sets.

LEV1ATHAN is an algorithm for generating a level-1 rooted phylogenetic network from a set of rooted triplets [5]. Specifically, it attempts to find a level-1 rooted phylogenetic network that contains as many of the input rooted triplets as possible. This problem is an NP-hard problem [5]. The algorithm by [6] can be used to find a level-1 or a level-2 rooted phylogenetic network which minimizes the number of reticulation nodes, if such a network exists. In [6] the authors also showed that for a dense set of rooted triplets τ, if τ is precisely equal to the set of rooted triplets that are contained in some rooted phylogenetic network, then they can construct such a rooted phylogenetic network with smallest possible level in time *O*(|τ|*^k^* ^+1^), where *k* is a fixed upper bound on the level of the network. In addition based on the ideas described in [6], for a given dense set of rooted triplets τ, the authors proposed the SIMPLISTIC algorithm which always returns some rooted phylogenetic network that contains τ. But it does not give any minimality guarantees.

In [7] the authors showed that given a dense set of rooted triplets τ and a fixed number *k*, it is possible to construct in time *O*(|τ |*^k^* ^+1^) a level-*k* rooted phylogenetic network that contains τ or decides that no such network exists.

In this paper we present a heuristic algorithm called *TripNet* for constructing rooted phylogenetic networks with the minimum number of reticulation nodes from an arbitrary set of rooted triplets. Despite of current methods that work for dense set of rooted triplets, a key innovation is the applicability of TripNet to non-dense set of rooted triplets.

In “unpublished data” the authors applied TripNet on both real and simulated data. Here TripNet algorithm is described in details, some theorems are proved, and one simulation is performed to show the accuracy of TripNet. Also TripNet is tested on the Yeast data. This paper is organized as follows. In section 2, first some definitions and notation are presented. Then we describe BUILD algorithm. Finally a new method called TCD, is introduced for constructing rooted triplets from (biological) sequences. In section 3 we compare TripNet with SIMPLISTIC on the triplets sets that are obtained from TCD method. Then we test TripNet on the Yeast data. In section 4 we discuss the performance of TripNet. In the last section the directed graph Gτ related to a set of triplets τ is introduced. Then we show that if either a set of triplets is obtained from a set of sequences using TCD method or a set of triplets is consistent with a tree, then *G*_τ_ is a DAG. This property has a key role in solving the Integer Programming system which is introduced in the remaining, in polynomial time. Then the concept of the height function of a rooted phylogenetic network is introduced, and an efficient method for obtaining a height function *h*_τ_ for a given set of rooted triplets τ is explained. It is shown that the condition of consistency of a rooted phylogenetic network *N* with the height function *h*_τ_ can be a good alternative for the condition of consistency of *N* with τ. To show this, firstly we define the Integer Programming system in such a way that its constraints intuitively force the consistency of *N* with τ. Secondly, we show that if τ is consistent with a tree *T*, then *T* is consistent with *h*_τ_ and *T* can be constructed using this height function. In the last section we present TripNet algorithm.

## Preliminaries

Here first we present some definitions and notation. Then we describe BUILD algorithm. Finally a new method called TCD, is introduced for constructing rooted triplets from a set of sequences.

### 2.1 Definitions and notation

Let *X* be a set of taxa. A *rooted phylogenetic tree* (*tree* for short) on *X* is a rooted unordered leaf labeled tree whose leaves are distinctly labeled by *X* and every node which is not a leaf has at least outdegree two. A *directed acyclic graph* (DAG) is a directed graph that is free of directed cycles. A DAG *G* is *connected* if there is an undirected path between any two nodes of *G*. It is *biconnected* if it contains no node whose removal disconnects *G*. A biconnected component of a graph *G* is a maximal biconnected subgraph of *G*. A *rooted phylogenetic network* (*network* for short) on *X* is a rooted DAG in which the *root* has indegree 0 and outdegree 2 and every node except the root satisfies one of the following conditions:

- It has indegree 2 and outdegree 1. These nodes are called *reticulation* nodes.
- It has indegree 1 and outdegree 2.
- It has indegree 1 and outdegree 0. These nodes are called leaves and are distinctly labeled by *X*.

A *reticulation leaf* is a leaf whose parent is a reticulation node. A network is said to be a *level-k network* if each of its biconnected components contains at most *k* reticulation nodes. A tree can be considered as a level-0 network.

**Figure 1:**
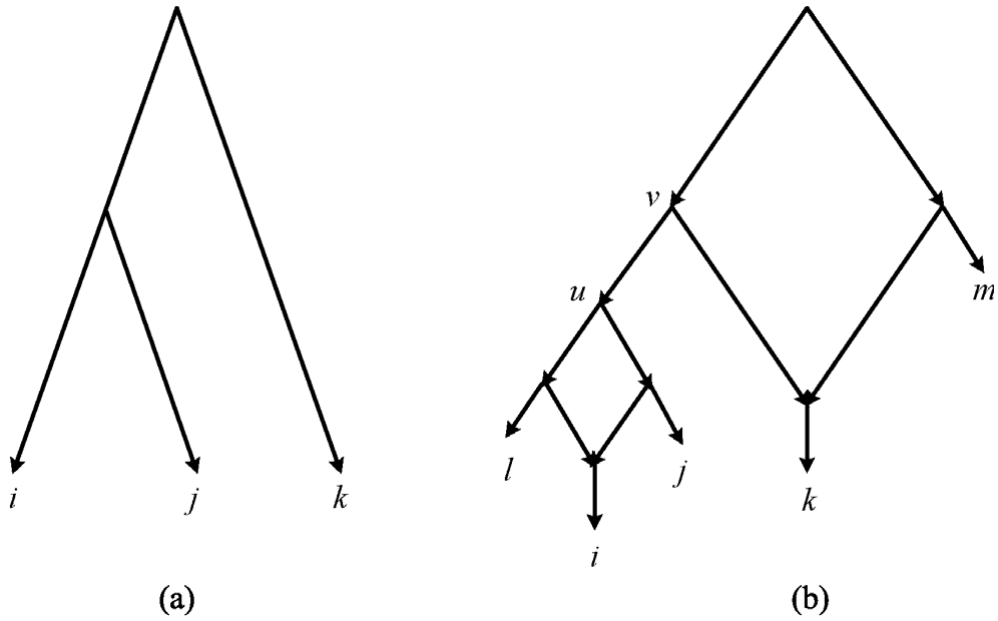
A triplet and a network consistent with it. (a) The triplet $ij|k$, (b) The triplet *ij*|*k* is consistent with the given network.

A *rooted triplet* (*triplet* for short) is a rooted binary unordered tree with three leaves. We use *ij*|*k* to denote a triplet with taxa *i* and *j* on one side and *k* on the other side of the root (Figure 1a). A set of triplets τ is called *dense* if for each subset of three taxa, there is at least one triplet in τ. A triplet *ij*|*k* is *consistent* with a network *N* or equivalently *N* is consistent with *ij*|*k* if the leaf set of *ij*|*k* is a subset of the leaf set of *N*, and *N* contains a subdivision of *ij*|*k*, i.e. if *N* contains distinct nodes *u* and *v* and pairwise internally node-disjoint paths *u* → *i*, *u* → *j*, *v* → *u* and *v* → *k*. Figure 1b shows an example of a network consistent with *ij*|*k*. A set τ of triplets is consistent with a network *N* if all the triplets in τ are consistent with *N*. We use the symbols τ(*N*) and *L_N_* to represent the set of all triplets that are consistent with *N* and the set of labels of its leaves respectively. For any set τ of triplets define *L*(τ) = 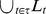. The set τ is called a set of triplets on *X* if *L*(τ) = *X*.

### 2.2 BUILD algorithm

Let τ be a set of triplets. BUILD is a top-down algorithm, constructs a tree consistent with τ if such a tree exists. The algorithm is guided by the Aho graph.

**Definition 1.** (Aho graph) Let *X* be a set of taxa and τ be a set of triples on *X*. The Aho graph AG(τ) = (*V*,*E*) associated with τ has node set *V* = *X* and any two nodes *i* and *j* are connected by an edge in *E* if and only if there exists a triplet *ij*|*k* ∈ τ [1].

**Figure 2:**
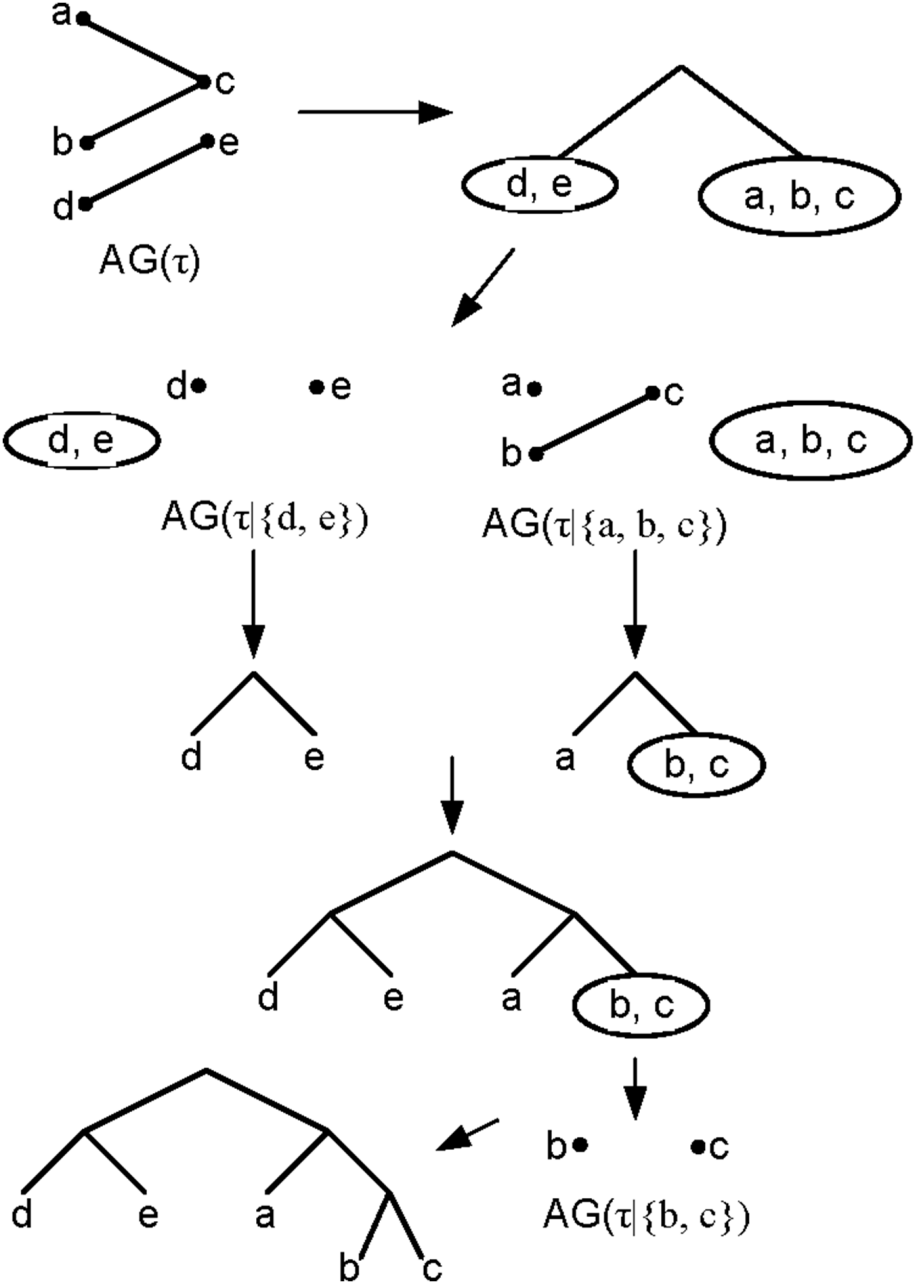
An example of BUILD algorithm for the given set {*bc *|*a*, *ac *|*d*, ***de*** **|** ***b*** **} of triplets.**

BUILD algorithm: Given a non-empty set of rooted triples τ on *X*, the aim is to construct a rooted phylogenetic tree *T* on *X* that is consistent with τ, if one exists. If AG(τ) has only one connected component, then the algorithm reports fail. Else, for each node set *U* of a connected component of AG(τ), determine the set τ|*_U_* which denotes the set of all triplets in τ whose leaves are in *U* and recursively compute the rooted phylogenetic subtree *T*(τ|*_U_*) which denotes the tree constructed with BUILD algortihm consistent with τ|*_U_*. Finally, create a root node *r* and combine all computed subtrees by connecting *r* to the root of each of them [1]. For an example see Figure 2.

### 2.3 Triplets construction method

There exist different methods like Maximum Parsimony or Maximum Likelihood for constructing triplets from (biological) sequences [6]. In this section a method for constructing triplets is presented. Suppose that *X* is a set of *n* taxa, and *D* = [*D_ij_*] be an *n×n* distance matrix on *X*. For each three taxa *i*, *j*, and *k* ∈ *X*, and the entries *D_ij_*, *D_ik_*, and *D_jk_*, we assign the triplet *ij*|*k* if *D_ij_* < min {*D_ik_*, *D_jk_*}. We name this method Triplets Construction with Distance; TCD for short. In this paper we use TCD method for constructing triplets.

## Results

In this section to show the performance of TripNet on the triplets sets which are obtained from TCD, we compare TripNet with SIMPLISTIC. Also we test TripNet on the Yeast data. It is the only published triplets data that are obtained from biological data.

### 3.1 Comparing SIMPLISTIC and TripNet

SIMPLISTIC is the only available software which has the ability to return some rooted phylogenetic network consistent with a given dense set of rooted triplets. But it does not give any minimality guarantees [6].

SplitsTree is a valuable tool for constructing an special kind of unrooted phylogenetic networks from different types of data as input. This program converts a given set of sequences *X* into a distance matrix *D_X_* to compute the resulting network. The distance matrix *D_X_* is reported as one of the output of SplitsTree [8].

Let 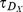 be the set of triplets that is obtained from *D_X_* using TCD, and consider it as the input for TripNet.

Note that 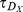 is not necessarily dense, since for some three taxa *i*, *j*, and *k* we might have 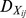 = 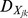
<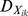. In this case one of the triplets *ij*|*k* or *jk*|*i* is assigned to *i*, *j*, and *k* to obtain a dense set of triplets 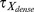 as the input of SIMPLISTIC. Also if 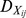 = 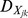 = 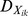, then randomly one of the three possible triplets related to *i*, *j* and *k* is assigned to them.

To perform the simulation we generate 160 different sets of sequences are generated using TREEVOLVE. TREVOLVE is a software which simulate the evolution of DNA sequences under a coalescent model [9]. TREEVOLVE contains many input parameters which one can adjust them. In this study we adjust the *Number of samples*, the *Number of sequences*, and the *Length of sequence*, and for the other parameters the default values are adjusted. In this study the *Number of sequences* is 10, 20, 30, and 40. For each input parameter the *Number of sequences* the *Length of sequence* is 100, 200, 300, and 400. For each case the *Number of samples* is set to 10.

In this study we run both methods on a PC with an Intel DuallCore processor running at 1.80 GHz.

We set the running time restriction 6 hours for methods. Let *N_finite_* be the set of networks for which the running time is less than 6 hours.

The results of the comparison between TripNet and SIMPLISTIC on the three most important parameters i.e. running time of both methods, number of the reticulation nodes and the level of the final networks, are shown in Table 1.

**Table 1:** SIMPLISTIC and TripNet network results.

Number of sequences	10	20	30	40
Number of samples	40	40	40	40
Number of the TripNet networks ∈ *N_finite_*	40	40	40	40
Number of the SIMPLISTIC networks ∈ *N_finite_*	40	38	13	0
TripNet avg runningtime for networks ∈ *N_finite_* (Sec)	1	1.75	200	775
SIMPLISTIC avg runningtime for networks ∈ *N_finite_* (Sec)	1	306	2675	-
TripNet avg number of reticulations for networks ∈ *N_finite_*	0.65	2.275	7.4	15.825
SIMPLISTIC avg number of reticulations for networks *N_finite_*	2.325	6.95	11.275	-
TripNet avg level for networks *N_finite_*	0.65	1.825	6.95	15.25
SIMPLISTIC avg level for networks *N_finite_*	2.05	4.2	6.95	-

160 different sets of sequences are generated using TREEVOLVE. the parameters *Number of samples*, the *Number of sequences*, and the *Length of sequence* are adjusted, and for the other parameters the default values are adjusted. *Number of sequences* is 10, 20, 30, and 40. For each input parameter the *Number of sequences* the *Length of sequence* is 100, 200, 300, and 400. For each case the *Number of samples* is set to 10. *N_finite_* is the set of networks for which the running time is less than 6 hours.

The results show that when the number of input taxa is 10, both methods always return a network in at most one second. For the number of input 20, in 5% of cases SIMPLISTIC returns no results in less than 6 hours. For the remaining 95% of the cases, the SIMPLISTIC running time is on average 306 seconds, while in all cases on average the TripNet running time is at most 2 seconds. But by increasing this parameter to 30, in 67.5% of the cases, SIMPLISTIC has not the ability to return a network in less than 6 hours. For the remaining 22.5% of the cases on average SIMPLISTIC outputs a network in 2675 seconds, while in all cases the TripNet running time is on average 200 seconds. Moreover when this parameter is set to 40, in all cases SIMPLISTIC fails to return any network in less than 6 hours, while on average TripNet outputs a network in 775 seconds. Totally for all 160 input triplets sets on average TripNet outputs a network in less than 250 seconds, while on average in 57% of the SIMPLISTIC networks which belong to *N_finite_*, the running time is near to 750 seconds.

Also the results show that in all cases the number of the reticulation nodes and the level of TripNet networks are less than SIMPLISTIC networks. Note that for the number of input 40, on average the number of the reticulation nodes and the level of the TripNet networks are 15.825 and 15.25, while for these data SIMPLISTIC can not return any network in less than 6 hours.

### 3.2 Yeast data

**Figure 3:**
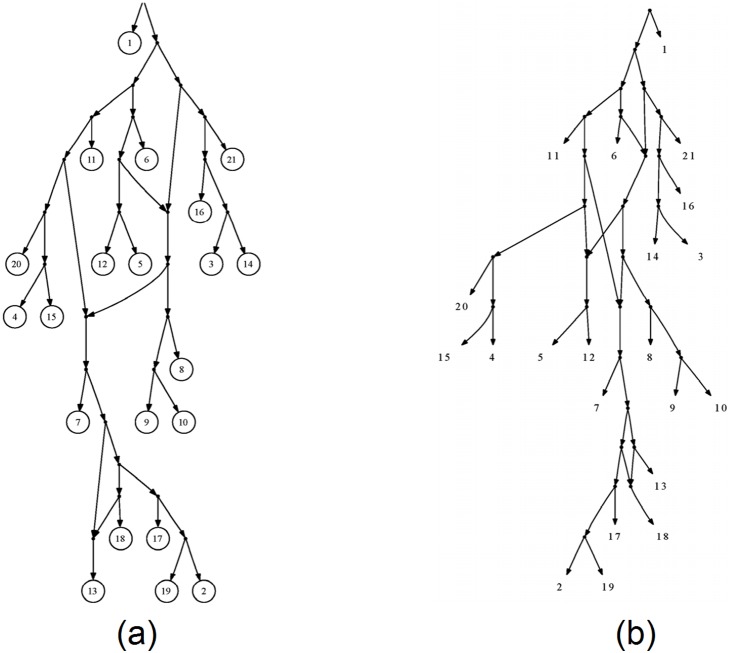
Resulting networks from Yeast triplets. (a) LEVEL2 algorithm result. (b) TripNet algorithm result.

The Yeast data is a dense set of triplets generated using real yeast data, obtained from the Fungal Biodiversity Center in Utrecht. This data set which contains information about 21 species is available online from (http://skelk.sdf-eu.org/level2triplets.html). Based on the algorithm developed in [10]. Steven Kelk has developed a software application, called LEVEL2, for constructing level-2 networks from dense sets of triplets. LEVEL2 is not applicable to general triplet sets and it produces a network only if there exists a level-2 network consistent with the input triplets. However, LEVEL2 has the advantage that it always produces the best possible network which also minimizes the number of reticulation nodes. LEVEL2 network for the Yeast data is a 21-leaf level-2 network which is given in Figure 3a [10]. As our only chance for comparing TripNet networks with the best possible networks we repeated the analysis of Yeast data using TripNet. The TripNet network for the Yeast dataset is given in Figure 3b. As one can see, TripNet produced a level-3 network which contains only one more reticulation node than the network obtained by LEVEL2. The running time of both algorithms is nearly one second.

## Discussion

In this paper we introduced TripNet which is the software that has the ability to return some network consistent with an arbitrary given set of triplets.TripNet and supplementary files are freely available for download at (www.bioinf.cs.ipm.ir/software/tripnet). Unlike previous methods which only work on dense triplet sets, our method works on any set of triplets. Some theorems were proved to clarify the rationale behind the steps of TripNet. In this paper the TCD method was introduced for constructing triplets. In order to study the performance of TripNet on the triplets that are obtained from TCD method we performed a simulation on 160 different sets of triplets, and compared TripNet with SIMPLISTIC.

The results showed that in all 160 cases TripNet outputs an appropriate network in an acceptable time, while just in 57.5% of these cases SIMPLISTIC has the ability to return some network in less than 6 hours. Also on average in all cases TripNet outperforms SIMPLISTIC on the number of the reticulation nodes, and the level of the output network.

Also by increasing the number of input taxa, the running time of SIMPLISTIC exceeds abruptly, such that for the input taxa 40, it could not return any network in less than 6 hours.

These results showed that for large size input data that are obtained from TCD method, SIMPLISTIC is not a practical method for constructing networks, while TripNet works well in all cases.

To establish the performance of TripNet on real datasets, we tested TripNet on Yeast data, and compared our results with those of LEVEL2. For Yeast data TripNet produced a level-3 network which contains only one more reticulation node than the optimal network obtained by LEVEL2. Both networks have the same clustering and represent the same evolutionary relationship between taxa. While TripNet has been designed for general triplet sets (not necessarily dense or consistent with a restricted level network), this example shows that the network produced by TripNet is very close to the best possible solution.

## Materials and Methods

In this section we prove some theorems to clarify the rationale behind the steps of TripNet. Then TripNet is presented in nine steps.

### 5.1 The directed graph related to a set of triplets and height function

Throughout this subsection we denote *i*, *j* by *ij* for short. Let τ be a set of triplets. Define *G*_τ_, the directed graph related to τ, by *V*(*G*_τ_) = {*ij*: *i*,*j* ∈ L(τ), i ≠ j} and *E*(*G*_τ_) = {(*ij*,*ik*): *ij*|*k* ∈ τ} ∪ {(*ij*,*jk*): *ij*|*k* ∈ τ}. In the following we present some basic properties of *G*_τ_.

In what follows the height function of a tree is introduced. Let 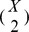 denotes the set of all subsets of *X* of size 2.

**Definition 2.** Let *X* be an arbitrary finite set. A function *h*: 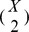 → 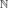 is called a *height function* on *X*.

Let *T* be a rooted tree with the root *r,* c*_ij_* be the lowest common ancestor of the leaves *i* and *j*, and *l_T_* denotes the length of a longest path starting at *r*.

**Definition 3.** The *height function* of *T, h_T_* is defined as *h_T_*(*i,j*)* = l_T_-d_T_*(*r,c_ij_*) where *i* and *j* are two distinct leaves of *T* (*d_T_*(*r,c_ij_*) denotes the length of the path between *r* and *c_ij_*).

Let *T* be a tree. The definition above implies that a triplet *ij*|*k* is consistent with *T* if and only if *h_T_*(*i*, *j*)<*h_T_*(*i*, *k*) or *h_T_*(*i*, *j*)<*h_T_*(*j*, *k*).

Let *X* = {*x*_1_, *x*_2_, …, *x_m_*} be a finite set, *D* be a distance matrix on *X*, and τ be the set of triplets on *X* that are obtained from TCD method using *D*. Let *G*_τ_ contains a cycle *x*_1_ *x*_ 2_ → *x*_2_ *x*_ 3_ → … → *x_n_*_−1_ *x_n_* → *x*_1_ *x*_ 2_. Then 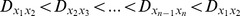, which is a contradiction. So *G*_τ_ is a DAG.

Moreover if τ is a triplet set consistent with a tree *T*, then *G*_τ_ is a DAG. This is so because if *G*_τ_ contains a cycle *x*_1_ *x*_2_ → *x*_2_ *x*_ 3_ → … → *x_n_*_−1_ *x_n_* → *x*_1_ *x*_ 2_, then *h_T_*(*x*_1_,*x*_2_) < *h_T_*(*x*_2_,*x*_3_) < … < *h_T_*(*x_n_*_−1_,*x_n_*) < *h_T_*(*x*_1_,*x*_2_), which is a contradiction.

The height function of a DAG is introduced as what follows.

Let τ be a set of triplets, *G*_τ_ be a DAG and 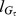 denotes the length of the longest path in *G*_τ_. Since *G*_τ_ is a DAG, the set of nodes with outdegree zero is nonempty. Assign 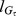
+1 to the nodes with outdegree zero and remove them from *G*_τ_. Assign 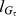 to the nodes with outdegree zero in the resulting graph and continue this procedure until all nodes are removed.

**Definition 4.** For any two distinct *i*, *j* ∈ *L*(τ), define 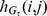 as the value that is assigned by the above procedure to the node *ij* and call it the *height function* related to *G*_τ_.

Let τ be a set of triplets that is consistent with a tree, and *T*_τ_ denotes the unique tree that is produced by BUILD algorithm. Then *G*_τ_ is a DAG and 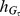 is well-defined. The following theorem represents an upper bound for 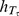 based on 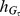.

**Theorem 1.** Let τ be a set of triplets that is consistent with a tree. Then 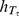 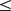 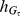.

**Proof.** The proof proceeds by induction on 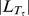. It is trivial when 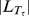 = 3. Assume that theorem holds when 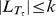. Let 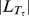 = *k*+1 and *T*_1_, *T*_2_, …, *T_m_* be *m* subtrees which are obtained from *T*_τ_ by removing its root. For each *i*, 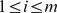, let 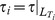, and *r_i_* be the root of *T_i_*. By the induction assumption for each *i*, 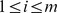,
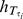
≤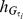. Moreover we conclude from BUILD algorithm that 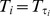, for 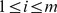. Thus 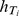 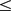 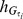, for 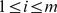. Also for *i*, 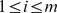
_,_ the maximum length of the longest path in *T_i_* is 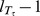. It means that for *i*, 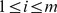, the maximum length of the longest path in 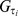 is at least 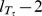. Therefore the length of the longest path in 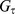
is at least 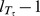. Let 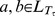. We have two cases.

**Case 1.** For some *i* and *j*, 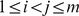, 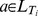 and 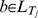. Since the outdegree of *ab* in 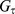 is zero and *c_ab_* = *r*, then 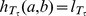
≤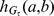.

**Case 2.** For some *i*, 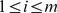, 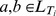. By the induction assumption 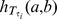
≤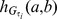 for *i*, 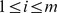. Therefore 
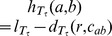
 = 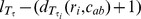 =  
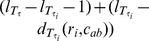
 = 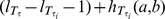
≤
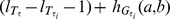
≤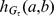. The last inequality is obtained by construction of 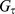 from 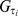 for *i*, 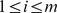.

So for each 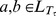, 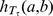
≤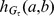 and the proof is complete.

**Figure 4:**
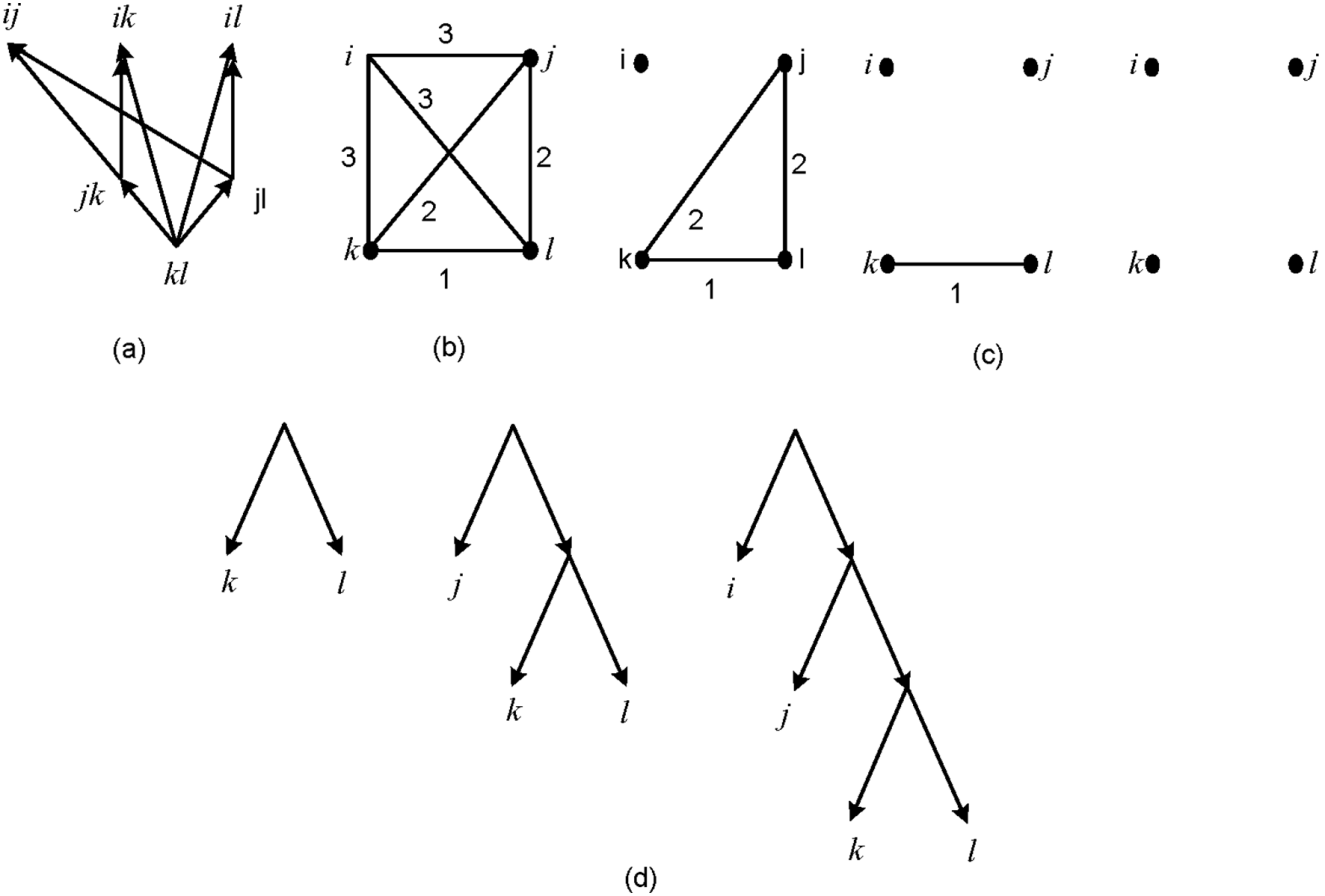
The steps of constructing *T*_τ_ from the given set τ = {*kl *|*j*, ***kl*** **|** ***i*** **,** ***jk*** **|** ***i*** **,** ***jl*** **|** ***i*** **}** **,** **using HBUILD.** (a) The graph *G*_τ_, (b) The graph (*G*,*h*), (c) Removing maximum weights from the graph (*G*,*h*), (d) Constructing *T*_τ_ using step c.

Now we describe an algorithm similar to BUILD algorithm, using height functions. We refer to this algorithm by *HBUILD*. Let *h* be a height function on *X*. Define a weighted complete graph (*G*,*h*) where *V*(*G*) = *X* and edge {*i*, *j*} has weight *h*(*i*,*j*). Remove the edges with maximum weight from *G*. If removing these edges results in a connected graph the algorithm stops. Otherwise, the process of removing the edges with maximum weight is continued in each connected component until each connected component contains only one node. At the end of this procedure one can reconstruct the tree by reversing the steps of the algorithm similar to BUILD algorithm (see Figure 4). The algorithm above decides in polynomial time whether a tree with height function *h* exists.

So if τ is a set of triplets which is consistent with a tree, then 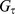 is a DAG and 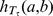
≤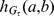 = h and HBUILD algorithm constructsa tree consistent with τ. Note that based on theorem 1 the tree that is produced by *HBUILD* is exactly *T*_τ._

The HBUILD tree is not necessarily a binary tree. To obtain a binary tree consistent with a set of triplets, we do the following procedure.

**Figure 5:**
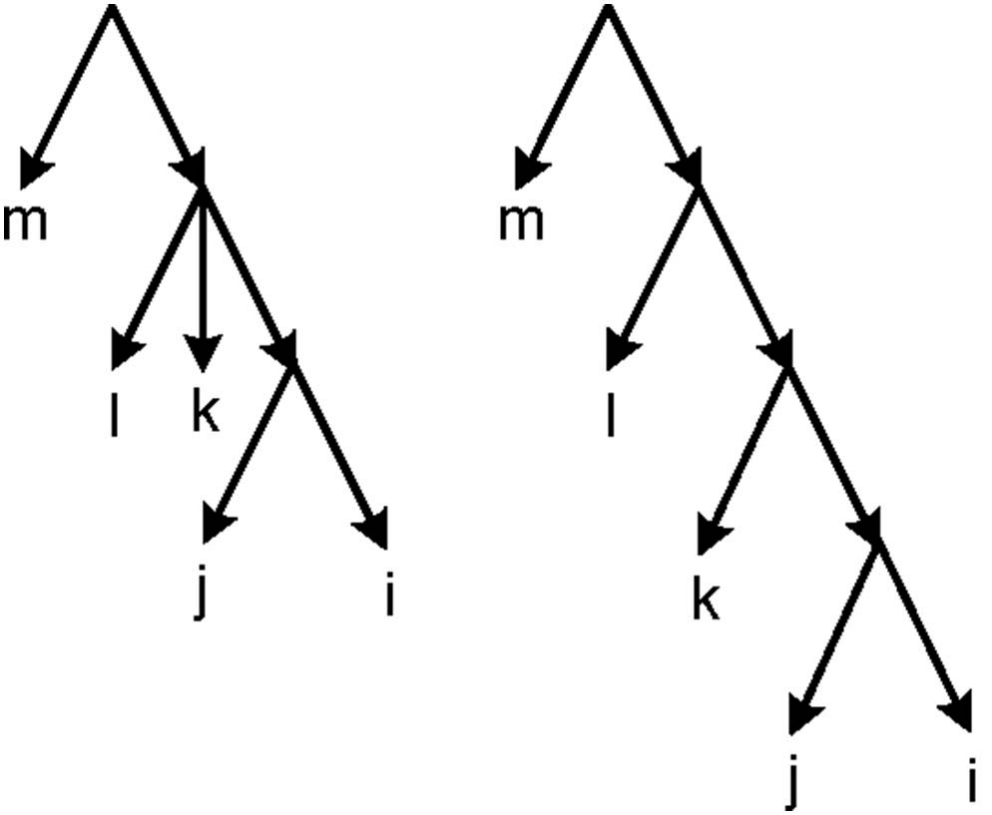
An example of binarization. The binary tree is a binarization of the non-binary tree.

Let *T* be a tree and *x* be a node of *T* with *x*_1_, *x*_2_, …, *x_k_*, 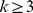 as its children. Consider a new node *y*. Construct 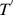 by removing the edges (*x*, *x*_1_), (*x*, *x*_2_), …, (*x*, *x_k_*_-1_) from *T* and adding the edges (*x*, *y*), (*y*, *x*_1_), (*y*, *x*_2_), …, (*y*, *x_k_*_-1_) to *T*. Continuing the same method for each node with outdegree more than 2 a binary tree is obtained, and call it a *binarization* of *T* (see Figure 5). Obviously, one can obtain different binarization of *T*. Let τ be a set of triplets that is consistent with a tree *T*_1_, and *T*_2_ be a binarization of *T*_1_. Then τ is consistent with *T*_2_.

In the remaining of this section we generalize the concept of height function from trees to networks. This generalization is not straightforward because the concept of (lowest) common ancestor of two leaves of a network is not well-defined. Let *N* be a network with the root *r* and *l_N_* be the length of a longest directed path from *r* to the leaves. For each node *u* consider *d*(*r*,*u*) as the length of the longest directed path from *r* to *u*. For any two nodes *u* and *v*, we call *u* an *ancestor* of *v*, if there is a directed path from *u* to *v*. If *u* is an ancestor of *v* then we say that *v* is *lower* than *u*. Let *i* and *j* be two leaves of *N*. *c* is called a *lowest common ancestor* of *i* and *j* in *N*, if *c* is a common ancestor of *i* and *j* and there is no common ancestor of *i* and *j* lower than *c*. For any two leaves *i* and *j*, let *C_ij_* denote the set of all lowest common ancestors of *i* and *j*.

**Definition 5.** For each pair of leaves *i* and *j*, define *h_N_*(*i*,*j*) =  *min*{*l_N_*-*d*(*r*,*c*): *c*∈*C_ij_*} and call it the *height function* of *N*.

**Figure 6:**
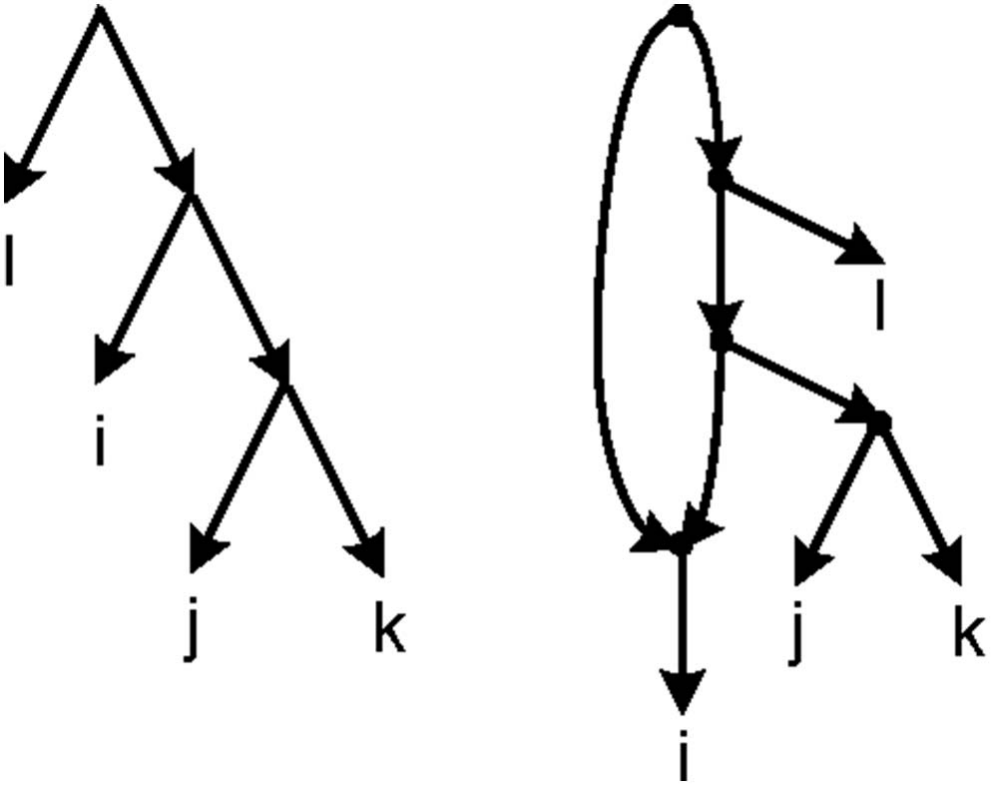
Two different networks with the same height function. For the given network *N* and tree *T*, *h_N_* = *h_T_* = *h*. *h*(*j*,*k*) = 1, *h*(*i*,*j*) = *h*(*i*,*k*) = 2 and *h*(*i*,l) = *h*(*j*,*l*) = *h*(*k*,*l*) = 3.

Obviously, every network *N* indicates a unique height function *h_N_*. But two different networks may have the same height function (see Figure 6).

In the following proposition we prove that for a given height function *h* there is a network *N* such that *h_N_* = *h*+1.

**Proposition 1.** Let *X* be an arbitrary finite set and *h* be a height function on *X*. Then there exists a network *N* not necessarily binary, such that its leaves are distinctly labeled by *X* and *h_N_* = *h*+1.

**Proof.** Let *X* = {*x*_1_, *x*_2_, …, *x_n_*} and *h_max_* = *max*{*h*(*x_i_*, *x_j_*): 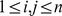}. Let *r* be the root of *N*,, and *X*′ = {*x*′_1_, *x*′_2_, …, *x*′_n_}. Consider *n* nodes that are distinctly labeled by *X*′ members. For each pair of nodes *x_i_* and *x_j_* with *h*(*x_i_*, *x_j_*)  =  *h_max_*, connect *x*′*_i_* and *x*′*_j_* to *r* by two paths of length *h_max_* which just are common in the root. For each pair of nodes *x_i_* and *x_j_* with *h*(*x_i_*, *x_j_*) < *h_max_*, consider a new node and connect *x*′*_i_* and *x*′*_j_* to this new node and connect this node to *r* by a path of length *h_max_*-*h*(*x_i_*, *x_j_*). For each node which is labeled by *x*′*_i_*, consider a new node as its child and label it by *x_i_*. The resulting network in which its leaves are distinctly labeled by *X* satisfies the condition *h_N_* = *h*+1.

Note that the network *N* which is constructed in the proof of Proposition 1 is not necessarily a rooted phylogenetic network. To construct a rooted phylogenetic network *N*′ from *N* in such a way that if a triplet is consistent with *N* then it is consistent with *N*′, do the following procedure. Replace each path in which all its inner nodes have indegree and outdegree one, with a path of length one. The method of constructing *N* shows that If there is a node *v* with indegree 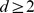, then it has just one child as a leaf. Let this child is labeled by *x*, 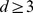 and its *d* parents are labeled by *x*_1_, *x*_2_, …, *x_d_*. Replace the edge which is connected to *x* with a path of length *d*-2 in such a way that its *d*-2 inner nodes from *v* to *x* are labeled with 1 to *d*-2. For each *i*, 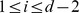 remove the edge *x_i_v* and connect *x_i_* to *i*. Do the binarization on the root. The resulting network *N*′ is consistent with all triplets which are consistent with *N*.

The following theorem shows relation between the height function of a network and a triplet consistent with it.

**Theorem 2.** Let *N* be a network, *i*, *j*, and *k* be its three distinct leaves. If *h_N_*(*i*, *j*) < *h_N_*(*i*, *k*) or *h_N_*(*i*, *j*) < *h_N_*(*j*, *k*) then *ij*|*k* is consistent with *N*.

**Proof.** Suppose that *h_N_*(*i*, *j*) < *h_N_*(*i*, *k*). Let *v_ij_*$ and *v_ik_* be common ancestors of *i*, *j* and *i*, *k* respectively, such that *h_N_*(*i*, *j*)  =  *l_N_*-*d*(*v_ij_*, *r*) and *h_N_*(*i*, *k*)  =  *l_N_*-*d*(*r*,*v_ik_*). Let *l_i_* and *l_j_* be two distinct paths from *v_ij_* to *i* and *j*, respectively. Let *l_k_* be an arbitrary path from *v_ik_* to *k*. If 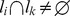 then it follows that 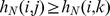 which is a contradiction. So *ij*|*k* is consistent with *N*.

**Figure 7:**
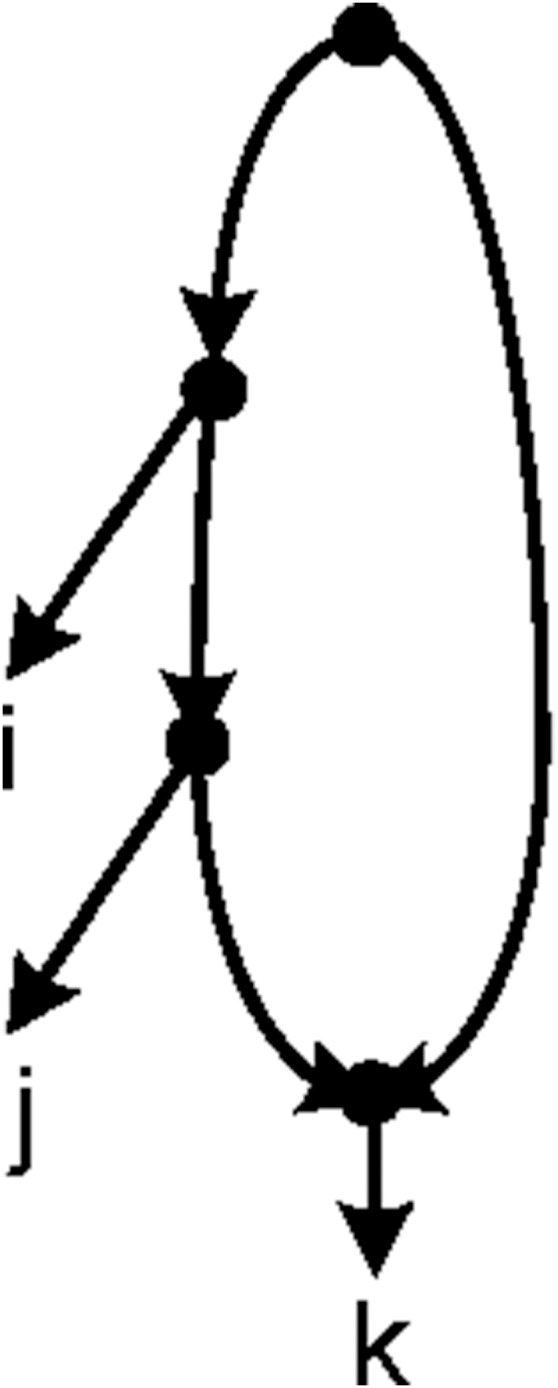
A counter example for the reverse of Theorem 2. *ij*|*k* is consistent with the given network, but *h*(*i*,*j*) = *h*(*i*,*k*) = 3 and *h*(*j*,*k*) = 2.

The reverse of the above theorem is not necessarily true. For example, consider the network of Figure 7. The triplet *ij*|*k* is consistent with it, but *h*(*i*,*j*) = *h*(*i*,*k*) = 3 and *h*(*j*,*k*) = 2.

The basic idea of TripNet algorithm is to find a height function as an intermediate computational step that yields the minimum amount of information required to construct the network from a set of triplets. So it is important to find a way for computing *h_N_* from a set of triplets. In the rest of this section we introduce a computational method for computing *h_N_* using Integer Programming. Let τ be a set of triplets with |*L*(τ)| = *n*. Inspired from the two inequalities that are the consequence of Definition 3 and Theorem 2, for each triplet *ij*|*k* ∈ τ, define two inequalities 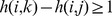 and 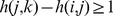. Since the number of variables in such inequalities are at most 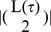, we obtain the following system of inequalities from τ.



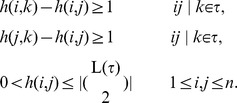



Let *s* be an integer. Define the following Integer Programming and call it IP(τ,*s*).



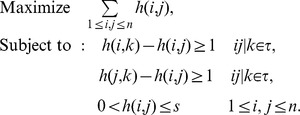
Intuitively if IP(τ,*s*) has a feasible solution, we expect that the optimal solution to this integer programming is an approximation of the height function of an optimal network *N* consistent with τ. The following theorems support this intuition.

**Theorem 3.** Let τ be a set of triplets. Then *G*_τ_ is a DAG if and only if for some integer *s*, the IP(τ,*s*) has a feasible solution. In this case the minimum number *s*, for which IP(τ,*s*) has a feasible solution, is 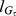
+1.

**Proof.** Let *G*_τ_ be a DAG. Without loss of generality assume that *G*_τ_ is connected.

The proof proceeds by induction on 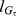. If 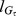 = 1 then obviously for *s* = 1, IP(τ,*s*) has no feasible solution and for each 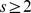, IP(τ,*s*) has a feasible solution. Assume that the theorem holds for 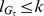. Suppose that τ is a set of triplets with 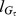 = *k*+1. Let *A* be the set of the terminal nodes of all longest paths in *G*_τ_. For each ij ∈ *A* there is some *x* ∈ *L*(τ) such that *ix*|*j* ∈ τ. Let *B* be the set of all such triplets and τ′ = τ\*B*. Apparently, *B*≠φ and the length of the longest path in *G*_τ_′ is *k*. By the induction assumption the minimum number *s* for which IP(τ′,*s*) has a feasible solution, is 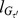 +1 = 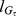. Consider IP(τ,
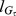
+1). Define *h*(*i*, *j*) = 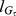
+1, for each ij ∈ *A* and *h*(*t*,*l*) = *h*′(*t*,*l*), for each *tl* 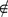 *A*. *h* is a feasible solution to IP(τ,
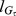
+1). Now if *s* is a solution for IP(τ,*s*) then *s*-1 is a solution for IP(τ′,*s*-1). So 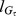
+1 is the minimum solution for IP(τ,*s*). Now suppose that τ is a set of triplets and for some integer *s*, IP(τ,*s*) has a feasible solution *h*. Assume that *G*_τ_ has a cycle 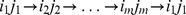. Corresponds to *C* we have inequalities 
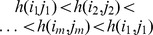
which is a contradiction and the proof is complete.

Let τ be a set of triplets that is consistent with a tree or constructed from a given set of taxa, using TCD method. It was shown that *G*_τ_ is a DAG and by Theorem 3, 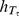 is a feasible solution to IP(τ,
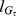
+1).

**Theorem 4.** Let τ be a set of triplets consistent with a tree. Then 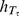 is the unique optimal solution to IP(τ,
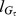
+1).

**Proof.** The graph *G*_τ_ is a DAG, since τ is consistent with a tree. So 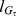 is well efined.

The proof proceeds by induction on 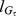. Without loss of generality assume that *G*_τ_ is connected. The theorem is trivial when 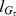 = 1. Let for each set of triplets consistent with a tree, 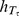 be the unique optimal solution to IP(τ,
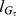
+1) where 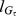 =  *k* 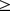
1. Suppose that τ is a set of triplets consistent with a tree and 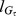 =  *k*+1. Let τ′ be the set of triplets which is introduced in the proof of Theorem 3. By the induction assumption 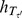 is the unique optimal solution to IP(τ′, 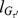
+1). By Theorem 3 the minimum *s* for which IP(τ, *s*) has a feasible solution is 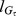
+1. Also 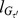
+1 = 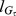. It follows that 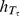 is the unique optimal solution to the IP(τ,
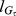
+1) and the proof is complete.

It is important to point out that the introduced target function of the above IP can be replaced with other appropriate target functions. But we use this special target function because it can be easily possible to find a solution for this IP in polynomial time when the input triplets are obtained from TCD method. Secondly using this target function, enable us to prove those above theorems which show the consistency of the result of the TripNet algorithm with a tree when there is a tree consistent with given triplets.

### 5.2 TripNet algorithm

Now we describe the TripNet algorithm in nine steps. In this algorithm the input is a set of triplets τ and the output is a network consistent with τ. Also if τ is consistent with a tree the algorithm constructs a binarization of *T*_τ_.

**Step 1.** In this step we find a height function *h* on *L*(τ). If *G*_τ_ is a DAG we set *G*′_τ_ =  *G*_τ_. If *G*_τ_ is not a DAG we remove some edges from *G*_τ_ in such a way that the resulting graph *G*′_τ_ is a DAG. Set *h* = 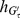.

If τ is obtained from a set of taxa using TCD method, then *G*_τ_ is a DAG. Removing minimum number of edges from a directed graph to make it a DAG is known as the *minimum Feedback Arc Set* problem which is NP-hard [11]. Thus we use the following heuristic method and try to remove as minimum number of edges as possible from *G*_τ_ in order to lose minimum information. First a cycle *C* is selected randomly. Let *C_max_* denote the set of nodes in *C* with the maximum degree. Remove an edge of *C* which one of its ends belongs to *C_max_*. This process continues until the resulting graph is a DAG. However, any such missing information will be recaptured in Step 9.

**Step 2.** In this step TripNet first apply HBUILD on *h*. If the result is a tree, TripNet constructs a binarization of this tree. Otherwise TripNet goes to Step 3.

Note that if τ is consistent with a tree, TripNet constructs a binarization of *T*_τ_.

**Step 3.** Remove all the maximum-weight edges from *G*. The process of removing all the maximum-weight edges from the graph continues until the resulting graph is disconnected.

In [3] and [4] the authors introduced the concept of *SN*-sets for a set of triplets τ. A subset *S* of *L*(τ) is an *SN-set* if there is no triplet *ij*|*k* ∈ τ such that *i* 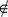 *S* and *j*, *k* ∈ *S*. In [4] it is shown that if τ is dense then the maximal *SN*-sets partition *L*(τ) and can be found in polynomial time. By contracting each of the *SN*-set to a single node and assuming a common ancestor for all of these leaves, the size of the problem is reduced. In these papers, for finding the maximal *SN*-sets in polynomial time, the authors use the high density of the input triplet sets. TripNet algorithm uses the concept of height function as an auxiliary tool to obtain *SN*-sets instead of the high density assumption.

**Step 4.** For each connected component obtained in Step 3 which is not an *SN*-set, we apply Step 3. This process continues until all of the resulting components are *SN*-sets. Let {*S*_1_, *S*_2_, …, *S_k_*} be the set of resulting *SN*-sets. If each *SN*-set contains only one node, HBUILD is applied and if the result is a tree TripNet constructs a binary tree and goes to Step 6. Otherwise TripNet goes to Step 5. If for some *i*, |*S_i_*|>1, contract each *S_i_* to a single node *s_i_* and set *S* = {*s*_1_, *s*_2_, …, *s_k_*}. Update the set of triplets by defining τ*_S_* = {*s_i_s_j_*|*s_k_*: if ∃ *xy*|*z* ∈ τ, *x* ∈ *S_i_*, *y* ∈ *S_j_* and *z* ∈ *S_k_*}. Constructs a weighted complete graph (*G_S_*, *w_S_*) with *V*(*G_S_*) = *S* and *w_S_*(*s_i_*, *s_j_*) = *min* {*h*(*x*, *y*): * x* ∈ *S_i_* and *y* ∈ *S_j_*}. Set (*G*, *w*) = (*G_S_*, *w_S_*) and TripNet goes to Step 3.

The following theorem is a consequence of the definition *SN*-set for (*G_S_*, *w_S_*).

**Theorem 5.** Applying Steps 3 and 4 on (*G_S_*, *w_S_*) and τ*_S_*, each resulting *SN*-set has one member.

**Proof.** Suppose that *S* = {*s*_1_, *s*_2_, *s*_3_, …, *s_r_*} is an *SN*-set in (*G_S_*, *w_S_*). Now assume that in the procedure of Step 3 by removing the edges with weight *l*, *S*_1_ separates from *S*_2_. Thus there exists *k* > *l* such that by removing the edges with weight at least *k* in (*G_S_*, *w_S_*), the connected component *S* separates from other components of *G_S_*. It means that by removing the edges with weight at least *k* in *G*, we obtain the *SN*-set 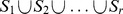 which is a contradiction.

In the next step the reticulation leaves are recognized using the following three criteria:

**Criterion I.** Let *m_i_* and *M_i_* be the minimum and maximum weight of the edges in (*G*,*h*) with exactly one end in *S_i_*. Choose the node with minimum *m_i_* and if there is more than one node with minimum *m_i_* then choose among them the nodes which has minimum *M_i_*. Let *R*_1_ denotes the set of such nodes.

**Criterion II.** Let *w_min_* =  *min* {*w*(*s_i_*,*s_j_*): 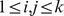}. In *G_S_* consider the induced subgraph on the edges with the weight *w_min_*. Choose the nodes of *R*_1_ with the maximum degree in this induced subgraph. Let *R*_2_ denotes the set of such nodes.

**Criterion III.** For each node *s* ∈ *R*_2_, remove it from *GS* and find *SN*-sets for this new graph using Steps 3 and 4. Let *n_s_* be the number of *SN*-sets of this new graph with cardinality greater than one. Choose the nodes in *R*_2_ with maximum *n_s_*. Let *R*_3_ denotes the set of such nodes.

We state an example to show the idea behind these three criteria.

Let τ = {*ij*|*l*, *jk*|*i*, *kl*|*j*, *kl*|*i*, *no*|*m*, *lo*|*k*, *jl*|*o*, *mn*|*l*, *mn*|*j*, *no*|*k*, *mo*|*i*, *jk*|*n*, *ij*|*o*, *ik*|*m*, *il*|*n*}.

**Figure 8:**
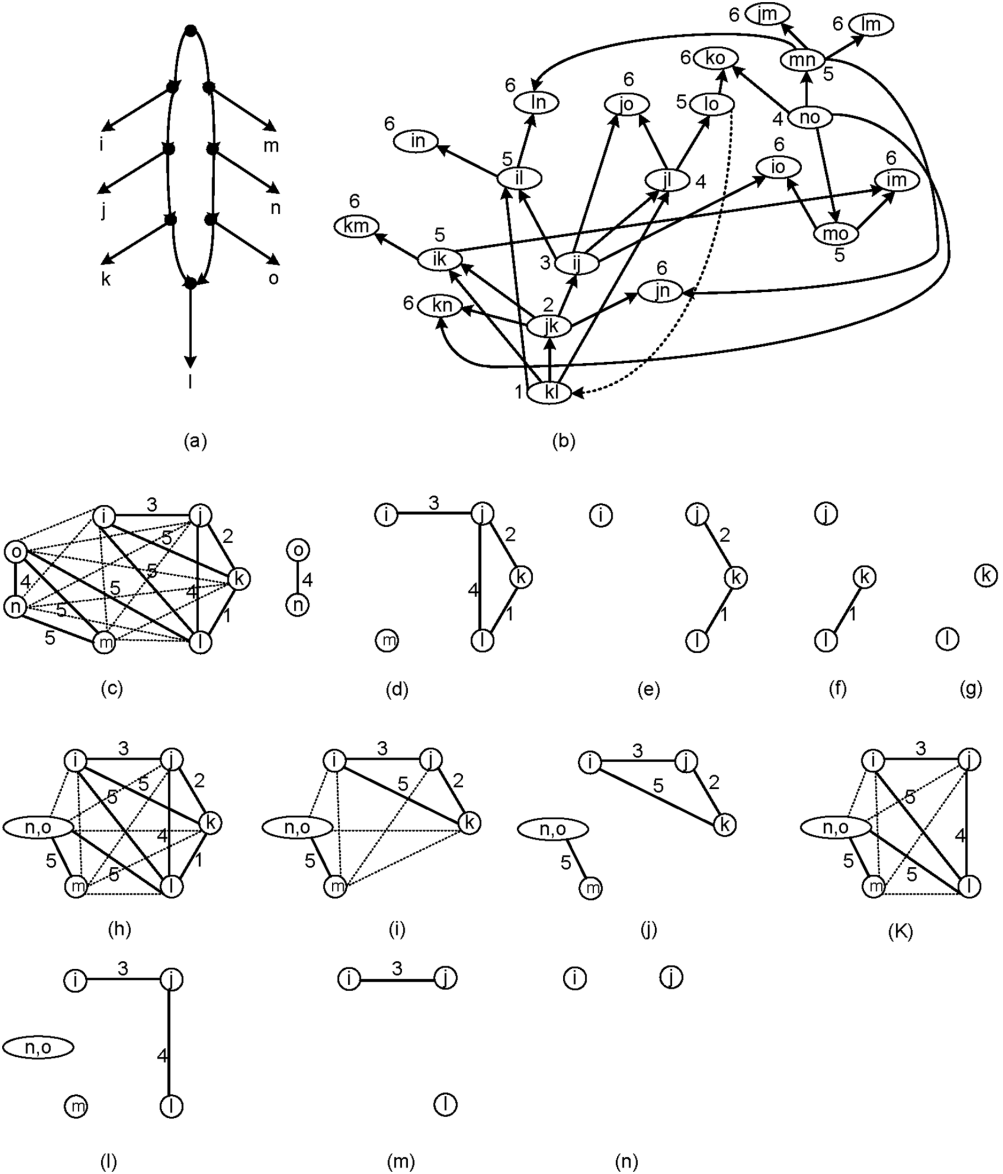
An example to show how TripNet works to find a reticulation leaf by applying step 5. Edges with weight 6 are shown by dotted lines. (a) τ = {*ij*|*l*, *jk*|*i*, *kl*|*j*, *kl*|*i*, *no*|*m*, *lo*|*k*, *jl*|*o*, *mn*|*l*, *mn*|*j*, *no*|*k*, *mo*|*i*, *jk*|*n*, *ij*|*o*, *ik*|*m*, *il*|*n*} is not consistent with a tree and is consistent with the given level-1 network, (b) G′_τ_ is obtained from G_τ_ by removing the dotted line, (c) Computing (*G*, *h*), (d) Remove edges with weights 6 and 5 from (*G*, *h*) to obtain *SN*-sets {*n*, *o*} and {*m*}, (e) Remove edges with weights 4 and 3 from the remaining graph to obtain *SN*-set {*i*}, (f) Remove edges with weights 2 from the remaining graph to obtain *SN*-set {*j*}, (g) Remove edges with weights 1 from the remaining graph to obtain *SN*-sets {*k*} and {*l*}, (h) Compute *G_S_*. both *SN*-sets {*k*} and {*l*} satisfy Criteria I and II, (i) Remove {*l*} from *G_S_*, (j) Remove edges with weights 6 from the graph of previous step to obtain *SN*-sets {*i*, *j*, *k*} and {*m*, *n*, *o*}, (k) Remove {*k*} from *G_S_*, (l) Remove edges with weights 6 and 5 from the graph of previous step to obtain *SN*-sets {*n*, *o*} and {*m*}, (m) Remove edges with weights 4 from the remaining graph to obtain *SN*-set {*l*}, (n) Remove edges with weights 3 from the remaining graph to obtain *SN*-sets {*i*} and {*j*}. The steps i to n shows that *l* is the reticulation leaf. In these steps criterion III is applied.

 τ is not consistent with a tree but it is consistent with the network *N* shown in Figure 8a. Obviously, *N* is an optimal network consistent with τ. In order to find *SN*-sets we construct *G*′_τ_ and (*G*, *h*), and find *SN*-sets from (*G*, *h*) using Steps 3 and 4 (Figures 8b to 8g). It follows that *S* = {{*i*}, {*j*}, {*k*}, {*l*}, {*m*}, {*n*, *o*}}. Now in *G_S_* (Figure 8h). we expect that the reticulation is in *R*_1_. In this example both *k* and *l* are in *R*_1_. Also we expect that if there is a reticulation leaf, it belongs to *R*_2_ which again both *k* and *l* are in *R*_2_. Now just *l* belongs to *R*_3_. Thus we consider *l* as the reticulation leaf (Figures 8i to 8n). Remove triplets from τ*_S_* which contain *l* and denote the new set of triplets by τ′*_S_*. Obviously τ′*_S_* is consistent with a tree. We add this reticulation leaf to a binarization of 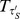 such that the resulting network is consistent with τ*_S_*. Note that if we consider each node except than *l* as the reticulation leaf then final network consistent with τ*_S_* has at least two reticulation leaves.

**Step 5.** In this step the reticulation leaf is recognized using three criteria. Do the criterion I. If |*R*_1_| = 1 then choose the node *x* ∈ *R*_1_ as the reticulation node. Otherwise if |*R*_1_|>1 do the criterion II. If |*R*_2_| = 1 then choose the node *x* ∈ *R*_2_ as the reticulation node. Otherwise if |*R*_2_|>1 do the criterion III. If |*R*_3_| = 1 then choose the node *x* ∈ *R*_3_ as the reticulation node. Otherwise if |*R*_3_|>1 then by the speed options we choose the reticulation node as follows.

**Slow.** Each node in *R*_3_ is examined as the reticulation leaf.

**Normal.** Two nodes in *R*_3_ are selected randomly and each of these two nodes is examined as the reticulation leaf.

**Fast.** One node in *R*_3_ is selected randomly as the reticulation leaf.

Let *x* be a node which is considered as a reticulation leaf. Remove *x* from *G_S_* and all of the triplets which contain *x* from τ_S_. Define *G* = *G* \ {*x*} and go to Step 3.

Note that for the Fast option the running time of the algorithm is polynomial.

For biological data almost always the criteria I and II find a unique reticulation leaf.

So on real data the running time of TripNet is almost always polynomial.

**Step 6.** Let *x*_1_, *x*_2_, …, *x_m_* be *m* reticulation leaves which are obtained in Step 5 with this order and *T* be the tree that is constructed in Step 4. Now add these *m* nodes in the reverse order to *T* as what follows. Let *e*_1_ and *e*_2_ be two edges of *T*. Consider two new nodes *y*_1_ and *y*_2_ in the middle of *e*_1_ and *e*_2_. Connect *y*_1_ and *y*_2_ to a new node *y*_3_ and connect the reticulation leaf *x_m_* to *y*_3_. Do this procedure for all pairs of edges and choose a pair such that the resulting network is consistent with maximum number of triplets in τ. Continue this procedure until all the reticulation nodes are added.

**Step 7.** For each *SN*-set *S_i_* and the set 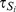
of triplets we run the algorithm again.

**Step 8.** Replace each *SN*-set in the network of Step 6 with its related network constructed in Step 7 to obtain a network *N*′.

Let τ′ ∈ τ be the set of the triplets which are not consistent with *N*′. For each pair of leaves *a* and *b* assume that τ′*_ab_* is the set of triplets in τ′ which are of the form *ab*|*c*. Consider the pair of leaves *i* and *j* such that τ′*_ij_* has the maximum cardinality. Assume that *p_i_* and *p_j_* are the parents of *i* and *j*, respectively.

**Step 9.** Create two new nodes in the middle of the edges *p_i_ i* and *p_j_ j* and connect them with a new edge. This new edge creates a reticulation node and all of the triplets in τ′*_ij_* will be consistent with the new network. All consistent triplets with the new network are removed from τ′ and this procedure will continue until τ′ becomes empty.

**Figure 9:**
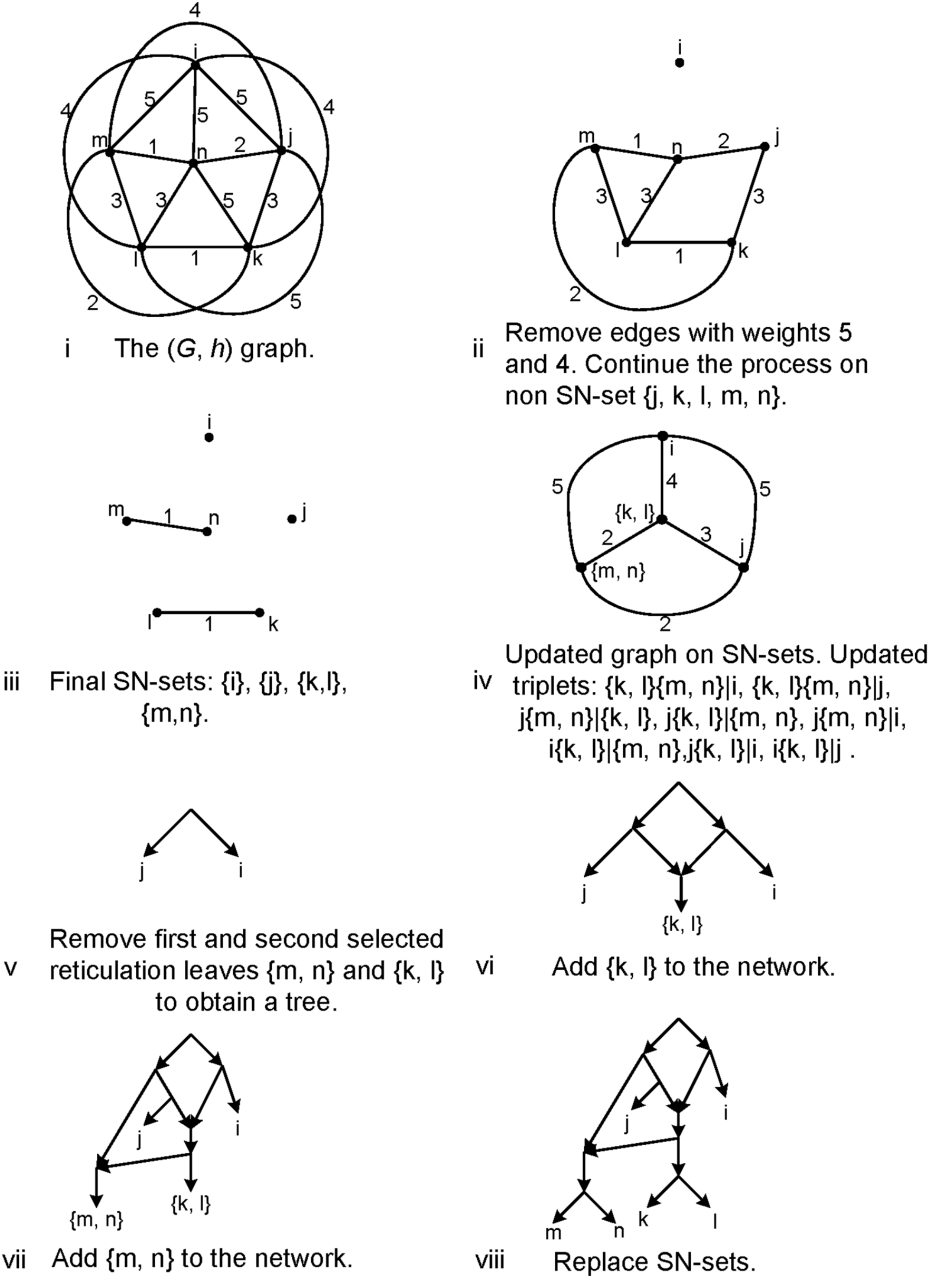
Steps of TripNet for input triplets: *jk *|*i*, *li *|*j*, *mj *|*i*, *jn*|*i*, *kl *|*i*, *ik *|*m*, *ik *|*n*, *lm*|*i*, *ln*|*i*, *mn*|*i*, *kl *|*j*, *km*|*j*, *jn*|*k*, *lm*|*j*, *jl *|*n*, *mn*|*j*, *kl *|*m*, *kl *|*n*, *mn*|*k*, *mn*|*l *}.

Figure 9 presents an example of the algorithm with all of its Steps.

## References

[1] Huson DH, Rupp R, Scornavacca C (2010) Phylogenetic Networks Concepts, Algorithms and Applications. Cambridge University Press.

[2] Aho AV, Sagiv Y, Szymanski TG, Ullman JD (1981) Inferring a tree from lowest common ancestors with an application to the optimization of relational expressions. SIAM J. Comp 10: 405–421.

[3] Jansson J, Nguyen NB, Sung WK (2006) Algorithms For combining rooted triplets into a galled phylogenetic network. SIAM Journal on Computing 35(5): 1098–1121.

[4] Jansson J, Sung Wk (2006) Inferring a Level-1 Phylogenetic Network from a Dense Set of Rooted Triplets. Theoretical Computer Science 363: 60–68.

[5] Huber K, Iersel LV, Kelk S, Suchecki R (2010) A Practical Algorithm for Reconstructing Level-1 Phylogenetic Networks. IEEE/ACM Transactions on Computational Biology and Bioinformatics. 10.1109/TCBB.2010.17 21393651

[6] Van Iersel L, Kelk S (2011) Constructing the simplest possible phylogenetic network from triplets. Algorithmica 60: 207–235.

[7] To TH, Habib M (2009) Level-k Phylogenetic Networks are Constructable from a Dense Triplet Set in Polynomial Time. In CPM09 5577: 275–288.

[8] Huson DH (1998) SplitsTree: analyzing and visualizing evolutionary data. Bioinformatics 14(10): 68–73. 9520503 10.1093/bioinformatics/14.1.68

[9] Grassly N, Rambaut A (1997) Treevole: a program to simulate the evolution of DNA sequences under different population dynamic scenarios. 1.3. Wellcome Centre for Infectious Disease, Department of Zoology, Oxford University, Oxford, UK.

[10] Van Iersel L, Keijsper J, Kelk S, Stougie L, Hagen F, et al (2009) Constructing level-2 phylogenetic networks from triplets. IEEE/ACM Transactions on Computational Biology and Bioinformatics 6(4): 667–681. 19875864 10.1109/TCBB.2009.22

[11] Karp R (1972) Reducibility among combinatorial problems. Proc. Sympos., IBM Thomas J. Watson Res. Center, Yorktown Heights, N.Y. 85–103.

